# Development of Graphene Quantum Dots-Based Optical Sensor for Toxic Metal Ion Detection

**DOI:** 10.3390/s19183850

**Published:** 2019-09-06

**Authors:** Nur Ain Asyiqin Anas, Yap Wing Fen, Nur Alia Sheh Omar, Wan Mohd Ebtisyam Mustaqim Mohd Daniyal, Nur Syahira Md Ramdzan, Silvan Saleviter

**Affiliations:** 1 Functional Devices Laboratory, Institute of Advanced Technology, Universiti Putra Malaysia, 43400 UPM Serdang, Selangor, Malaysia; 2 Department of Physics, Faculty of Science, Universiti Putra Malaysia, 43400 UPM Serdang, Selangor, Malaysia

**Keywords:** graphene quantum dots, metal ion, optical sensor, surface plasmon resonance

## Abstract

About 71% of the Earth’s surface is covered with water. Human beings, animals, and plants need water in order to survive. Therefore, it is one of the most important substances that exist on Earth. However, most of the water resources nowadays are insufficiently clean, since they are contaminated with toxic metal ions due to the improper disposal of pollutants into water through industrial and agricultural activities. These toxic metal ions need to be detected as fast as possible so that the situation will not become more critical and cause more harm in the future. Since then, numerous sensing methods have been proposed, including chemical and optical sensors that aim to detect these toxic metal ions. All of the researchers compete with each other to build sensors with the lowest limit of detection and high sensitivity and selectivity. Graphene quantum dots (GQDs) have emerged as a highly potential sensing material to incorporate with the developed sensors due to the advantages of GQDs. Several recent studies showed that GQDs, functionalized GQDs, and their composites were able to enhance the optical detection of metal ions. The aim of this paper is to review the existing, latest, and updated studies on optical sensing applications of GQDs-based materials toward toxic metal ions and future developments of an excellent GQDs-based SPR sensor as an alternative toxic metal ion sensor.

## 1. Introduction

As early as 1947, graphene has attracted the attention of many scientists. Unfortunately, most of the discoveries of graphene back then were only theoretical and unnoticeable. The actual name of “graphene” was given by Mouras et al. in 1987. In fact, graphene was only discovered again in 2004 when two physicists at the University of Manchester, Andre Geim and Konstantin Novoselov, decided to use sticky tape to peel off thin layers from a slab of graphite. Eventually, they got a layer that was just one atom thick, which then triggered a swamp of more experimental work of graphene and its potential applications.

Graphene is a thin, tightly packed layer of six carbon atoms that is bonded together in a hexagonal honeycomb lattice shape with a thickness of about 0.345 nm [1]. The physical shape of graphene also has many properties in terms of strength, electricity, and heat conduction, which generated enormous excitement because of its advantageous properties. However, graphene itself has a poor dispersion in solvents and aggregation. This means that it can be turned into many other forms such as colloidal, quantum dots, nanoribbons, and nanoplatelets. Furthermore, as a zero-band-gap semiconductor, the luminescence is almost impossible to observe, hindering the direct application of graphene. Therefore, the band gap can be manipulated by controlling the size, shape, and distribution of graphene nanostructures [2,3].

Since its discovery in the early 1980s, quantum dots (QDs) having a size ranging from 1 to 10 nm have attracted vast attention from researchers. QDs exhibit quantum phenomena that produce remarkable advantages in optical properties because of their size. It is a well-known fact that on excitation, the intensity and energy of emitted light will be higher when the size of QDs becomes smaller. QDs can be derived from semiconductors, metals, or carbon-based materials such as cadmium selenide quantum dots, cadmium sulfide quantum dots, graphene quantum dots, and carbon quantum dots. Compared to other QDs, graphene quantum dots (GQDs) attract more attention as sensitive analytical sensors with properties such as low toxicity, excellent biocompatibility, and highly solubility in various solvents [4].

**Figure 1:**
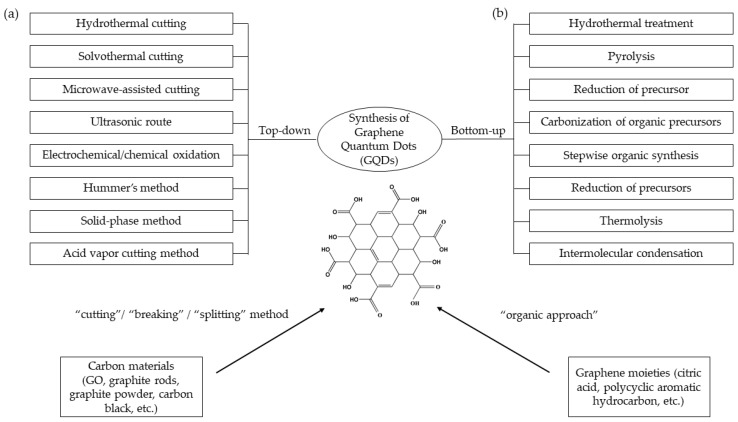
Representation of graphene quantum dots (GQDs) synthesized through (**a**) top–down methods and (**b**) bottom–up methods.

One way to obtain graphene-based fluorescent materials is to convert two-dimensional (2D) graphene sheets into zero-dimensional (0D) GQDs. GQDs exhibit extraordinary properties due to quantum confinement and the edge effect [5]. GQDs can be obtained either by cutting, splitting, or breaking down carbonaceous materials such as graphene oxide (GO), carbon black, carbon nanotubes, carbon fibers, and graphite rods; these are called the “top–down” methods [6,7,8]. Another method is through organic approaches of graphene moieties having few carbon atoms, which are called “bottom–up” methods [9,10]. The methods to synthesize GQDs are presented in Figure 1. Both methods have their own pros and cons. For the “top–down” method, it is much simpler compared to the “bottom–up” method. However, it has a lack control of size, morphology, and physical features of GQDs [11]. Changing the size of GQDs may affect the photoluminescence, band gap, and electron transfer ability of GQDs.

In order to confer such properties for a particular and desirable application, the synthesis of GQDs-based materials using different starting materials and methods can be controlled. For example, the large surface-to-volume ratio of GQDs is required to build a sensor, since it speeds up the absorption of target molecules. Such morphological and chemical control from different fabrication protocols affect the performance of sensors. As a result, the sensitivity, selectivity, biocompatibility, and limit of detection differ [12].

Due to the outstanding optical, electrical, mechanical, and thermal properties of GQDs, it has been one of the most popular choices to incorporate with sensors to detect toxic metal ions. Comparisons between different research studies for each type of semiconductor quantum dots have been made to highlight GQDs’ performance as sensors. This article comprehensively reviews the most recent trends in GQDs-based optical sensors and attempts at sensing toxic metal ions.

## 2. Incorporation of Graphene Quantum Dots with Optical Sensor for Toxic Metal Ion Detection

With the vast development of the world in many aspects including industrial and agricultural activities, the possibility for improper discharge of the pollutants into the environment also increases [13]. Consequently, environmental pollution has become one of the most critical problems in the world, especially water pollution caused by toxic metal ions. Water plays an important role throughout daily life. Therefore, we need clean and healthy resources of water. Examples of toxic metal ions can be found in water, including Pb^2+^, Hg^2+^, Fe^3+^, Cu^2+^, Cd^2+^, Co^2+^, Ni^2+^, Al^3+^, and Ag^+^. Some of these metal ions become toxic when they form poisonous solution compounds. They are non-degradable and do not decompose at all. Therefore, they continuously accumulate in the soil and subsequently in human bodies [14].

Due to awareness of these issues, there has been increasing activity in the field of toxic metal ions sensing until today. With the highly developed technologies, many variations of sensing strategies incorporated with different materials have been constructed to detect toxic metal ions. To date, many traditional methods have been widely used for the detection of metal ions such as atomic absorption spectroscopy (AAS) [15], anodic stripping voltammetry (ASV) [16], and plasma atomic emission spectroscopy [17]. They are accurate and sensitive. However, these methods suffer from limitations such as requiring expensive instruments and complicated sample treatments, as well as being time-consuming and highly destructive.

Other commonly modern methods used to detect toxic metal ions present in the environment and biological specimens include electrochemical [18,19] and electronic analyses [20]. However, the main drawbacks of the methods are low selectivity, instability, complex on-site sampling, and less compatibility in an aqueous environment. On the other hand, optical methods attracted the researchers’ attention to be used in toxic metal ions sensing, because this method is highly compatible in an aqueous environment, low cost, simple, fast, efficient, and highly sensitive and selective.

In this context, an optical sensor can be defined as a device that is able to give optical information such as absorbance, reflectance, or fluorescent emissions, and their change in intensity and quenching efficiency resulting from the interaction of materials and metal ions. The properties are measured in ultraviolet (UV), visible, or near infrared (NIR) ranges. Therefore, common optical methods that have been applied are fluorescent, electrochemiluminescence (ECL), photoluminescent (PL), colorimetric, and surface plasmon resonance (SPR) sensing. A comparison of the advantages and disadvantages of several optical sensors for heavy metal ions are summarized in Table 1.

As a new promising carbonic nanomaterial with a lot of outstanding advantages, GQD opened up a new field for the development of excellent sensors. Inspired by the advantages of optical methods, many works on the integration of GQDs with the above-named optical methods have been successfully done to effectively detect toxic meal ions. Therefore, the focus here is to review the latest and updated studies for optical sensing based on GQDs categorized by different toxic metal ions. The review includes both the single-shot testing (probe) and continuous testing (sensor) of toxic metal ions.

### 2.1. Ferric Ion (Fe^3+^)

In the environment and biological systems, Fe^3+^ is one of the most abundant and essential metal ions. Too much or a lack of Fe^3+^ can bring disadvantages to human beings. Insufficient Fe^3+^ ions can cause anemia, affecting the synthesis of hemoglobin, and restrict the delivery of oxygen to cells, which results in lethargy, low work performance, and decreased immunity. On the other hand, excess amounts of Fe^3+^ ions in a living cell can cause severe diseases such as hepatitis, organ dysfunction, chromatosis, and even cancers [21]. Several optical methods using GQDs have been reported to detect Fe^3+^.

The very first work for the optical detection of Fe^3+^ using GQDs-based material was reported by Zhou et al. in 2013. Due to the previous studies that revealed the special coordination between Fe^3+^ and phenolic hydroxyl groups, they insisted using pure green-emitting PL GQDs (P-GQDs) as a fluorescent probe for the sensitive detection of Fe^3+^. The GQDs were synthesized through the carbonization of pyrene as a precursor followed by hydrothermal reduction with hydrazine hydrate, which then produced P-GQDs. Even at an early stage, they managed to achieve a low limit of detection with a value of 5 nM [22].

The next year, Ju and Chen (2014) prepared nitrogen-doped GQDs (N-GQDs) via the hydrothermal treatment of hydrazine with GQDs synthesized by the pyrolysis of citric acid. Then, it was used as a fluorescent probe because they believed it was filled with oxygen-rich functional groups on its surface. Owing to both dynamic and static quenching processes, the PL of N-GQDs quenched linearly with increasing Fe^3+^ concentration within a wide range of 1 µM to 1945 µM and the detection limit was found to be 90 nM [23].

Ananthanarayanan et al. (2014) in the same year synthesized BMIM^+^-functionalized GQDs (BMIM^+^-GQDs) through electrochemical cutting of three-dimensional (3D) graphene using (1-butyl-3-methylimidazoliumhexafluorophosphate (BMIMPF_6_) as electrolyte and used it to detect Fe^3+^. They stated that there was high binding affinity between Fe^3+^ and the imidazole ring of BMIM^+^. They also believed that the fluorescence was quenched, resulting from the induced aggregation of GQDs where Fe^3+^ acts as coordination sites that bridge the BMIM^+^-GQDs together. A linear response between fluorescence quenching and Fe^3+^ concentration was obtained in a wide range up to 80 µM with a detection limit of 7220 nM [19].

The fabrication of nitrogen-doped GQDs (N-GQD) was done by Xu et al. (2014) using the acid vapor cutting method of metal–organic framework (MOF) derived carbon. Then, it was applied as a sensing probe to selectively detect Fe^3+^ in real water samples. As an explanation, when the complexation between Fe^3+^ and N-GQDs formed, the photo-induced charge transfer process leads to fluorescence quenching. The N-GQDs showed sensitive response to Fe^3+^ in a wide range of 1 µM to 70 µM with a detection limit of 80 nM [24].

Instead of using the “top–down” method [22], Tam et al. (2014) synthesized nitrogen-doped GQDs (N-GQDs) through the “bottom–up” hydrothermal method, which changed the electronic structure of GQDs. They successfully produced a green, inexpensive N-GQDs, which was then used for the determination of Fe^3+^. According to this study, the existence of hydroxyl groups on the surface and edges of N-GQDs forming complexes with the Fe^3+^ contributed to the mechanism of detection. This coordination restrained the recombination excitons, thereby quenching on the PL of GQDs. This system can detect Fe^3+^ as low as 1000 nM with Fe^3+^ concentrations ranging from 1 µM to 500 µM [25].

At the same time, Li et al. (2014) developed an efficient fluorescent probe for the detection of Fe^3+^ in serum samples using sulfur-doped GQDs (S-GQDs) prepared by the one-step electrolysis of graphite in sodium p-toluenesulfonate aqueous solution. This incorporation developed the coordination of Fe^3+^ with phenolic hydroxyl groups at the edges of S-GQDs, therefore quenching the PL of GQDs. A linear relationship was observed in the difference of fluorescence intensities against Fe^3+^ concentration raised to 0.7 µM with the detection limit of 4.2 nM [26].

Still in 2014, Li et al. attempted to use a single-step thermolysis process using glycine as a source for both carbon and nitrogen in order to produce nitrogen-doped and amino acid-functionalized GQDs (NA-GQDs). They found that the intensity of fluorescence decreased with increasing Fe^3+^ concentration within 0.5 µM to 500 µM. They wrote that Fe^3+^ has higher thermodynamic affinity compared to other metal ions, thus fastening the chelating process with nitrogen atoms. The obtained limit of detection of Fe^3+^ was 100 nM [27].

A year later, Xu et al. (2015) successfully synthesized GQDs containing rich oxygen-containing groups through the HNO_3_ vapor cutting method using a recyclable ordered mesoporous SiO_2_(SBA-15) template. Then, it was used for the optical detection of Fe^3+^. The PL intensity of the GQDs quenched slowly with the addition of Fe^3+^ in the range of 3 µM to 60 µM with a limit of detection of 300 nM. Upon the addition of other metal ions, Fe^3+^ gave the lowest ratio of intensity, which was probably due to the specific coordination between Fe^3+^ and phenolic hydroxyl groups. Furthermore, the Fe-GQDs complexation formed helped the photo-induced charge transfer from GQDs to Fe^3+^, therefore quenching the fluorescence [28].

An innovative work by Xu et al. (2015) showed the development of a masking-agent free dual-channel fluorescence response for toxic metal ions. This was by far the first work that used the dual-excitation GQDs-based pyrolysis of citric acid. In this study, the synthesized GQDs emitted blue PL under two different excitation wavelengths. They also used two channels; one responded to Fe^3+^ while Hg^2+^ exclusively quenched the other. The linear relationship between fluorescence intensity and Fe^3+^ concentration varied from 10 µM to 200 µM, in which the detection limit of 10,000 nM was observed for the first channel used to detect Fe^3+^ [29].

Another work by Guo et al. (2015) reported on the turn-on fluorescent sensing of Fe^3+^ based on rhodamine B derivative-functionalized GQDs (RBD-GQDs). First, electrochemical exfoliation and the acidic oxidation of a graphite rod was carried out in order to produce GQDs. Then, an acylation reaction proceeded to synthesize RBD-GQDs. The improvement of RBD’s sensitivity, photostability, biocompatibility, and solubility in water was also observed when incorporated with GQDs. The probe showed a detection limit of 20 nM. The difference in fluorescence intensity displayed a good linear relationship, with the Fe^3+^ concentration within the range of 0 to 1 µM. Interestingly, the fluorescent probe was further used to detect Fe^3+^ in cancer stem cells where the calculated limit of detection was also found to be 20 nM [30].

In 2016, Zhang et al. presented a simple step to obtain blue-emitting GQDs via the microwave-assisted pyrolysis of aspartic acid and an NH_4_HCO_3_ mixture. Then, synthesized GQDs were used as a fluorescent probe to sense Fe^3+^ due to the gained knowledge about good binding affinity between Fe^3+^ and hydroxyl groups. Fe^3+^ showed an obvious change in fluorescence intensity compared to other metal ions. Zhang et al. (2016) wrote that this was due to the Fe^3+^-GQDs complex, which resulted in the electron transfer to Fe^3+^ d-orbitals. The measured detection limit was 260 nM with good linear relationship between fluorescence intensity and Fe^3+^ concentration up to 50 µM [31].

Ligninsulfonate/GQDs (SL/GQDs) were prepared by Xu et al. (2016) for the sensitive and selective detection of Fe^3+^. The pyrolysis of citric acid producing GQDs was done, followed by the hydrothermal treatment of GQDs in an SL/NaOH system to synthesize SL/GQDs. SL at the edge of GQDs plays a remarkable role in the system, where they act as a fluorescence enhancer and chelator for Fe^3+^ detection. The SL/GQDs have high sensitivity toward Fe^3+^ due to the static quenching between them, which quenched the PL. A good linear correlation with PL intensities was observed over 0.005 µM to 500 µM of Fe^3+^ concentration. It is worthy to note that the sensor achieved a lowest limit of Fe^3+^ detection, which was 0.05 nM [32].

In the same year, Zhang and Gan developed a solid fluorescent sensor based on GQDs/polystyrenic anion-exchange resin (GQDs/PS-AER) to detect Fe^3+^. The acid oxidation of graphite produced GQDs, which then adsorbed by PS-AER to tailor GQDs/PS-AER. Interestingly, the sensor could be recovered by adding ethylenediaminetetraacetic acid (EDTA), which was followed by separating Fe^3+^ via filtration. With the increasing concentration of Fe^3+^ between 1–7 µM, the PL intensity was linearly decreased, and the calculated detection limit was 650 nM. As in previous works, GQDs/PS-AER enriched with hydroxyl groups on its surface encourage coordination to occur, resulting in charge transfer from GQDs/PS-AER to Fe^3+^ [33].

The strong affinity of dopamine toward Fe^3+^ made Chowdhury and Doong (2016) use dopamine-functionalized GQDs (DA-GQDs) as the fluorescent probe to detect Fe^3+^. The DA-GQDs were synthesized through the pyrolysis of citric acid and covalent conjugation with dopamine. They achieved a linear relationship between fluorescence intensity and the concentration of Fe^3+^ within the range of 0.02 µM to 2 µM and with a detection limit of 7.6 nM. They explained that the fluorescence quenching was caused by the oxidation of catechol moiety in the Fe–dopamine complex to the o-semiquinone of dopamine [34].

The acylation and amination reaction of graphene oxide (GO) nanosheets were performed by Ma et al. (2016) to synthesize amino acid-modified GQDs. They were named as l-alanine modified GQDs (AL-GQDs), asparagine-modified GQDs (AS-GQDs), and glycine-modified GQDs (GL-GQDs). Then, the prepared GQDs were used as fluorescent probes for sensing Fe^3+^. Among all three, AL-GQDs showed prominent sensing ability where obvious fluorescence quenching was observed upon the addition of Fe^3+^ due to the coordination of Fe^3+^ and carboxylic acid and amide groups of GQDs. A linear range of 0.05 µM to 200 µM with the lowest detection limit of 50 nM was obtained for AL-GQDs [35].

In 2017, a study on the optical sensor based on GQDs for the detection of Fe^3+^ was further developed. For example, Xuan et al. functionalized GQDs synthesized through the thermal pyrolysis of citric acid with D-penicillamine (DPA) or L-penicillamine (LPA), and found that the fluorescence of DPA-GQD was stronger and more sensitive toward Fe^3+^. The results verified that one of the significant factors that influence optical characteristics is the configuration of functional groups. The addition of Fe^3+^ concentration in the range of 0.004 mM to 1.8 mM leads to a decrease in fluorescence intensity, thus confirming that Fe^3+^ could quench DPA-GQD. Similar to any other studies, they wrote that this was due to the coordination formed between Fe^3+^ and functional groups on DPA-GQD. The limit of detection was calculated to be 1200 nM [36].

A sensor for Fe^3+^ detection in a water sample using boron-doped GQDs (BGQDs) was developed by Chen et al. in 2017. A simple electrolysis method in borax aqueous solution was first conducted to synthesize the BGQDs. They found that BGQDs are very sensitive toward Fe^3+^ compared to other concurrent metal ions, proving the high selectivity of Fe^3+^. The BGQDs’ fluorescence was quenched completely upon the addition of Fe^3+^. A linear relationship between fluorescence intensity and Fe^3+^ concentration within 0.01 µM to 100 µM was obtained. The system can also detect Fe^3+^ as low as 5 nM in real water samples [21].

A simple hydrothermal treatment of glutathione conducted by Xu et al. (2017) successfully produced two kinds of GQDs. In the presence and absence of Ag^+^, they obtained N-GQDs and SN-GQDs, respectively. Since the thiol-free N-GQDs gave a fast response toward Fe^3+^, they were then used as a fluorescent probe for Fe^3+^ sensing. The fluorescence intensity decreased with increasing concentrations of Fe^3+^ over the range of 50 µM to 2000 µM, and the limit of detection was calculated to be 50 nM [37].

Shen et al. (2017) innovatively synthesized new nitrogen and sulfur co-doped GQDs (N, S-GQDs) by a single-step, bottom–up molecular fusion of 1,3,6-trinitropyrene, thiourea, N,N-dimethylformamide (DMF), and sodium hydroxide in a hydrothermal process. The synthesized S,N-GQDs were further used as fluorescent probe to selectively detect Fe^3+^, Cu^2+^, and Ag^+^ in a mixture simultaneously, which depends on the masking agent used. For Fe^3+^, they used cysteine as a masking agent to isolate other metal ions, and observed a linear correlation between fluorescence intensity and concentration ranging from 0.01 µM to 25.0 µM and the lowest limit of detection was 8 nM, compared to Cu^2+^ and Ag^+^ [38].

In another study by Xia et al. (2017), free and solidified N, S-doped GQDs (N, S-GQDs) have been synthesized via a one-step solvent-free method (intermolecular dehydration and intermolecular condensation) using citric acid as precursors and L-cysteine as the dopant. The doping of GQDs improved the photoluminescence characteristics and influenced the modulation of chemical and electronic properties, which encouraged the coordination interaction between Fe^3+^ and phenolic hydroxyl groups on the edge of N, S-GQDs. For that, it made an excellent fluorescent probe for the detection of Fe^3+^. A linear range of 0.01 µM to 3 µM with a detection limit of 3.3 nM was also obtained [39].

Zhu et al. (2017) produced bare GQDs that served as a fluorescent probe for the detection of Fe^3+^. To prepare the GQDs, carbon black was oxidized with nitric acid. Similar to previous reports, Fe^3+^ formed a unique coordination with the phenolic hydroxyl groups on the GQDs’ surface to bridge neighboring GQDs together, thus triggering the fluorescence quenching. A linear relationship was observed between the fluorescence intensity of GQDs and Fe^3+^ concentration, which increased to 60 μM, and the limit of detection obtained was 450 nM [40].

Later, Wang et al. (2018) used rice husk as a raw material to synthesize GQDs based on a simple one-step one-pot hydrothermal method, and termed it as RH-GQDs. Then, the selective quenching properties of RH-GQDs were tested with several metal ions. However, Fe^3+^ showed an obvious quenching of luminescence compared to other metal ions. They also wrote about the Fe-GQDs complexes that promote charge transfer between them. Based on the plotted graph, a linear relation was observed between PL response and Fe^3+^ concentration raised to 10 mM, and the calculated detection limit equaled 5.8 nM [41].

Next, for the first time, Gao et al. in 2018 used the dual mode method (fluorometric and colorimetric method) for the detection of Fe^3+^ using N, Fe co-doped GQDs (N, Fe-GQDs). N, Fe-GQDs were fabricated through the hydrothermal reaction of ammonium iron (Ⅲ) citrate as the carbon source. For fluorescence behaviors, the fluorescence intensity of N, Fe-GQDs was quenched in the presence of Fe^3+^, which was probably due to the effective chelation interaction of Fe^3+^ with the functional groups of N, Fe-GQDs. The limit of detection was 3210 nM within a linear range of 10 to 110 μM. When using the colorimetric method, a remarkable increase in absorbance peak (color changes) was observed with increasing Fe^3+^ concentrations up to 450 μM. The detection limit was 1340 nM [42].

Presently, Wang et al. synthesized yellow-emitting amino-functionalized GQDs (af-GQDs) via chemical oxidation and amino-hydrothermal using high-softening point asphalt and ammonia as a precursor and nitrogen source, respectively. They successfully enhanced the fluorescence of GQDs with the addition of amide and amino groups. The af-GQDs were also served as fluorescent sensing probes to detect Fe^3+^. The ratio of fluorescence intensity displayed a good linear relation with Fe^3+^ concentrations between 0–50 µM. The calculated value for the limit of detection of Fe^3+^ was 0.51 nM [43]. Table 2 summarizes and compares the incorporation of GQDs in various optical methods for ferric ion detection.

### 2.2. Mercury Ion (Hg^2+^)

The high affinity for thiol groups in proteins and enzymes resulted in the toxicity of the Hg^2+^, which then leads to cells’ dysfunction [44]. Over some period of time, Hg^2+^ will eventually accumulate in the body, causing digestive, kidney, and neurological disease [45]. With the increasing concern over mercury in the environment and its harmful effects especially toward human health, many researchers have taken a step forward to build optical sensors based on GQDs to detect Hg^2+^ for the aforementioned advantages of GQDs to enhance the sensitivity of the sensors.

Chakraborti et al. first demonstrated the detection of Hg^2+^ using a fluorescent sensor based on GQDs in 2013. They prepared the GQDs first by modifying the carbonization degree of citric acid followed by dispersion into alkaline solution. Then, it was tested with several metal ions and surprisingly, only Hg^2+^ quenched the emission completely. Moreover, the fluorescence can be recovered with the addition of EDTA. The quenching fluorescence property was due to the adsorption of Hg^2+^ on the surface of GQDs, while the recovering process was due to the strong formation of the Hg^2+^–EDTA complex. The obtained value for the limit of detection of Hg^2+^ was 3360 nM [46].

Later in 2014, a study on florescent GQDs nanoprobes for the detection of Hg^2+^ was reported. Wang et al. prepared GQDs via a simple ultrasonic method and further explained the mechanism of the GQDs’ fluorescence quenching by Hg^2+^. Briefly, the electron transfer process happened upon the addition of Hg^2+^, which promotes non-radiative electron/hole recombination annihilation. Another explanation was that the prepared GQDs enriched with carboxylate groups exhibited special affinity with Hg^2+^, causing the aggregate-induced quenching of GQDs. Under optimum conditions, a linear range between 0.8–9 µM with a detection limit of 100 nM was obtained [47].

The study on the optical detection of Hg^2+^ based on GQDs was further developed in 2015. One of them was a study by Li et al. that synthesized GQDs through the pyrolysis of citric acid, and used it as a fluorescent probe for the detection of Hg^2+^. In the presence of Hg^2+^, the fluorescence quenching of GQDs was observed in accordance with the charge transfer mechanism reported in most studies. Excitingly, the fluorescence of GQDs was recovered upon the addition of certain amounts of cysteine, which encouraged them to develop dual sensors for Hg^2+^ and cysteine. GQDs were quenched linearly, with a Hg^2+^ concentration ranged from 1 nM to 50 nM with a calculated detection limit of 0.439 nM [48].

A simple method for the detection of Hg^2+^ in Hela cells via fluorescence sensing based on DNA-modified GQDs (DNA-GQDs) rich with thymine was also reported by Zhao et al. (2015). GQDs were first prepared via the hydrothermal reaction of graphite powder followed by a modification reaction with DNA, producing DNA-GQDs. The fluorescence quenching mechanism was attributed to the electron transfer process of DNA-GQDs. This resulted from the binding of Hg^2+^ with thymine bases, which led to the formation of non-radiative T–T mismatch hairpin structure. The change in fluorescence intensity of DNA-GQDs and Hg^2+^ concentration showed a linear relationship over the range 0.001 µM to 10 µM. The calculated limit of detection for Hg^2+^ was equal to 0.25 nM [49].

Shi et al. (2015) in the same year disclosed a simple strategy to prepare oxygen-rich nitrogen-doped GQDs (N-OGQDs) for the detection of Hg^2+^. The N-OGQDs were prepared through a simple one-pot solid-phase synthesis using citric acid (CA) as the carbon source and 3,4-dihydroxy-L-phenylalanine (L-DOPA) as the N source. When Hg^2+^ was added, it formed complexes with O atoms on the surface of N-OGQDs, forming strong affinity and causing a non-radiative electron transfer from excited states of N-OGQDs to the d orbital of Hg^2+^, and thus quenching the PL. The system exhibited a detection limit of 8.6 nM with a linear range of 0.04 µM to 6 µM [50].

A ratiometric fluorescent chemosensor based on GQDs (GQDs-SR) was developed for the intracellular imaging of Hg^2+^ in 2015 by Liu et al. Basically, the rhodamine derivative (energy acceptor) was conjugated onto the GQDs (energy donor) prepared from GO via Hummers method to produce GQDs-SR. This combination sensor provides the efficient sensing of Hg^2+^ where the presence of Hg^2+^ induced a ring-opening reaction of the spirolactam rhodamine (SR). Within 0.6 µM to 12 µM of Hg^2+^ concentration, the ratio of emission intensity was linearly decreased. The detection limit of Hg^2+^ in this work was found to be 230 nM [51].

Hua et al. (2015) prepared GQDs-based core–satellite hybrid spheres and further served such spheres as a ratiometric fluorescent probe for visualizing Hg^2+^. The pyrolyzing of citric acid was done in order to synthesize GQDs. The hybrid nanoparticles consist of red-emitting CdTe QDs (internal fluorescence material), silica surface (reaction sites), and blue-emitting GQDs (the outer layer that interacts with Hg^2+^). Upon the addition of Hg^2+^, the fluorescence emission of GQDs was quenched, with gradual colors changing from blue to red, resulting from a strong affinity between Hg^2+^ and the carboxylic and hydroxyl groups in GQDs. A linear relationship in the range of 0.01 µM to 22 µM with a detection limit of 3.3 nM was obtained [52].

Another study served green-emitting cysteine-functionalized GQDs (cys-GQDs) as a fluorescent probe for Hg^2+^ detection. Initially, Tam et al. (2015) synthesized cys-GQDs through the carbonization of citric acid, followed by the functionalization of L-cysteine via a hydrothermal process. Over the range of 1 µM to 500 µM of Hg^2+^ concentration, the fluorescence intensity changes of cys-GQDs were linearly decreasing, as caused by the electron transfer process resulting from the complexation of cys-GQDs and Hg^2+^. The sensor was able to detect Hg^2+^ as low as 20 nM [53].

There were vast and growing studies related to the optical detection of Hg^2+^ based on GQDs later in 2017. For example, Achadu and Nyokong showed the development of a novel PL nanoprobes for Hg^2+^ detection based on thymine-appended zinc phthalocyanine coordinated to pristine GQDs (GQDs-T-ZnPc). They prepared GQDs using GO by Hummers method and modified ZnPc with thymine to obtain T-ZnPc. Then, both T-ZnPc and GQDs were ultrasonicated to produce GQDs-T-ZnPc. The fluorescence emissions of GQDs were quenched upon conjugation with T-ZnPc. However, with the addition of Hg^2+^, the fluorescence managed to be restored, and was thus named as a “turn ON” detection of Hg^2+^ due to the specific affinity between thymine and Hg^2+^. Surprisingly, the system was able to detect Hg^2+^ as low as 0.05 nM within a linear concentration range of 0.1 nM to 20 nM [54].

A work by Xiaoyan et al. (2017) reported the synthesis of valine-functionalized GQDs (Val-GQDs) via the thermal pyrolysis of citric acid and valine. As a result, Val-GQDs showed a more sensitive response toward Hg^2+^ with the existence of valine moieties. In the presence of Hg^2+^, the fluorescence intensity of Val-GQDs was obviously quenched, which was attributed to the introduction of nitrogen atoms of valine into GQDs and resulting in a stronger interaction between Val-GQDs and Hg^2+^ to form complexes. The fluorescent quenching efficiency also decreased linearly with the increasing Hg^2+^ concentration between 0.8–1000 nM. The calculated detection limit was 0.4 nM [55].

Another one of the “turn ON” nanoprobes for the detection of Hg^2+^ was once again developed by Achadu and Nyokong (2017). This time, they used sulfur-containing phthalocyanines (Pcs) incorporated with pristine GQDs (termed as GQDs-Pcs). GQDs prepared by the hydrothermal reaction of GO were ultrasonicated with Pcs, forming GQDs-Pcs. In the presence of Pcs, the π–π stacking interaction that formed resulted in the quenching of fluorescence emission. However, the fluorescence was recovered upon the addition of Hg^2+^. This was due to the strong binding affinity of Hg^2+^ to the sulfur on Pcs, thus interrupting the π–π stacking interaction, which was subsequently named “turn ON” fluorescence for Hg^2+^ detection. The calculated limit of detection was the lowest by far with a value of 0.12 nM, and a linear range of 0.5 nM to 50 nM was also obtained [56].

Still in 2017, Alvand and Shemirani synthesized a multifunctional nanocomposite material, Fe_3_O_4_@SiO_2_@GQDs, by simply coating the GQDs-based pyrolysis of citric acid onto the surface of amine-functionalized Fe_3_O_4_@SiO_2_ nanospheres. The synthesized nanocomposite showed excellent performance for the detection of Hg^2+^. The fluorescence quenching efficiency of Fe_3_O_4_@SiO_2_@GQDs decreased linearly with increasing Hg^2+^ concentration from 0.1 µM to 70 µM, while the calculated limit of detection equals 30 nM [57].

Based on the fluorescence quenching of polyethyleneimine-functionalized GQDs (PEI-GQDs) upon π–π interaction or electrostatic attraction with mercaptopyridine-substituted zinc phthalocyanine (Pc-Au@Ag), Achadu and Nyokong (2017) once again developed a sensing platform for Hg^2+^ detection. The synthesis of PEI-GQDs followed previous methods, but used PEI instead of Pcs [54]. The fluorescence of PEI-GQDs was almost completely quenched upon the coordination with hte Pc-Au@Ag conjugate. Then, it was restored in the presence of Hg^2+^ before quenching back with biothiols addition. Thus, it was named an “off–on–off” process. A linear relationship between the ratio of fluorescence intensity and Hg^2+^ concentration was obtained within the range of 0.5 nM to 25 nM, while the value of the measured detection limit was 0.25 nM [58].

Next, Amini et al. (2017) synthesized GQDs via thermal pyrolysis with citric acid before functionalizing the surface with monoethanolamine (MEA). The MEA-GQDs showed high sensitivity toward Hg^2+^, which made them an excellent candidate as a fluorescent probe. Through increasing Hg^2+^ concentration, the fluorescence emission of MEA-GQDs gradually decreased due to the coordination of Hg^2+^ on the surface of MEA-GQDs, which led to a charge transfer process. A linear relationship was obtained between the change in fluorescence intensity and Hg^2+^ concentration ranging from 0.05 µM to 5 µM. The detection limit value of 10 nM was obtained. The fluorescence can be recovered with ethyl xanthate [59].

Still in the same year, Anh et al. fabricated N, S-co-doped GQDs (N, S-GQDs) by a one-pot hydrothermal treatment of citric acid and thiourea, and used it as a fluorescent probe for the sensitive and selective detection of Hg^2+^ in water. They explained that the S atoms served as active sites for Hg^2+^ coordination, while the N atoms enhanced the fluorescence yield of N, S-GQDs. The addition of Hg^2+^ gradually quenched the fluorescence of N, S-GQDs. A linear range of 0.05 µM to 15 µM was obtained, and the system can detect Hg^2+^ as low as 0.14 nM [60].

Manganese ion (Mn^2+^)-bonded nitrogen-doped GQDs (Mn(II)-NGQDs) were successfully produced by Yang et al. in 2018 through the one-pot hydrothermal carbonization of glycine, sodium citrate, and Mn^2+^. The Mn(II)-NGQDs displayed high sensitivity and selectivity toward Hg^2+^, and thus were used as a fluorescent probe for Hg^2+^. The developed sensor can detect Hg^2+^ up to 3.5 µM with a detection limit of 0.34 nM. They stated that the fluorescence quenching of Mn(II)-NGQDs was due to the static quenching process by Hg^2+^ [61].

Later, Ping et al. (2018) conducted a two-step thermal pyrolysis to synthesize pentaethyleneheaximine and D-penicillamine co-functionalized GQDs (PEHA-GQD-DPA) using citric acid, PEHA, and DPA as starting materials. The composition showed much improvement in the fluorescence emission since PEHA-GQD-DPA was enriched with amino, hydroxyl, and carboxyl groups, which promote the combination with Hg^2+^ via coordination bonds that formed the Hg-PEHA-GQD-DPA complex. Owing to that mechanism, the fluorescence intensity was quenched upon the addition of Hg^2+^ with a concentration ranging from 0.1 nM to 200 µM. This group successfully obtained a lower detection limit value of 0.046 nM. Additionally, they also found that the fluorescence can be restored by adding glutathione [62].

Su et al. carried out work on a fluorescent sensor for Hg^2+^ based on nitrogen-doped GQDs (N-GQDs) in 2018. They synthesized N-GQDs through a hydrothermal process that included citric acid in ammonia solution and served it as a fluorescent probe. The electron transfer process effectively occurred between Hg^2+^ and functional groups that were present on the surface of N-GQDs, thus forming N-GQDs/Hg^2+^ complexes. With the concentration of Hg^2+^ increased from 0.02 µM to 1 µM, the fluorescence intensity ratio decreased linearly, and the limit of detection measured for this work was 4.7 nM [63].

In 2019, a series of novel nitrogen and sulfur co-doped GQDs (N, S/GQDs) was used as a fluorescence probe for the detection of Hg^2+^. The work by Qu et al. (2019) initially started with the synthesis of N, S/GQDs via facile and green pyrolysis using citric acid and D-penicillamine as the carbon source and doped molecules, respectively. The doping encouraged the coordination between Hg^2+^ and the residual group on N, S/GQDs, hence improving the sensitivity toward Hg^2+^. The fluorescent sensor based on N, S/GQDs showed a linear correlation over Hg^2+^ concentration ranging from 0.9 nM to 30 nM with a limit of detection value of 0.69 nM [64].

Since the existence of N atoms can reduce the ability to form a stable complex between interfering cations and carboxyl groups, Yang et al. (2019) decided to synthesize rhodamine B assisted GQDs (RhB-GQDs) via a hydrothermal method using citric acid as the carbon source and rhodamine B and ethylenediamine as the nitrogen source, subsequently further using it as a fluorescent probe for the detection of Hg^2+^. Consequently, the fluorescence quenching happened even with the addition of a low concentration of Hg^2+^ due to the strong affinity of Hg^2+^ to amino and carboxyl on the surface of RhB-GQDs. A good linearity was obtained between the fluorescence quenching ratio, and the Hg^2+^ concentration increased to 10 nM with a calculated limit of detection value of 0.16 nM [65].

Recently, a work by Tang et al. (2019) successfully achieved the lowest limit of detection value for Hg^2+^ detection with a value of 0.00248 nM using an electrochemiluminescence (ECL) sensor. A poly(5-formylindole)/reduced graphene oxide (P5FIn/erGO) nanocomposite and Au nanoparticle-linked GQDs-DNA (GQDs-DNA-AuNP) were used as the ECL substrate and signal probe, simultaneously. The pyrolysis of citric acid was first done to obtain GQDs. Then, single-stranded DNA was introduced to the amino and sulfhydryl groups at each end of the GQDs and AuNP, respectively. In this work, the signal amplification capability was enhanced by the presence of AuNP, which increased the load of the GQDs. The sensor had a wide linear range of 0.01 nM to 100 nM [66]. The detection of mercury ions using different GQDs-based optical sensors is presented in Table 3.

### 2.3. Lead Ion (Pb^2+^)

Continuous exposure to Pb^2+^, even at a very low concentration, will have deleterious effects on human health and the environment. Exposure to Pb^2+^ can affect the reproductive, renal, hematopoietic, and central nervous system mainly by increased oxidative stress. One study also shows that the heartbeat frequency of a guinea pig increased when exposed to Pb^2+^ [67,68]. Thus, here are some studies related to optical detection based on GQDs of Pb^2+^.

A GQDs-based material was first used in 2013 by Qi et al. for the detection of Pb^2+^. They ran the fluorescent detection of Pb^2+^ using 3,9-dithia-6-monoazaundecane (DMA) functionalized GQDs (GQD-DMA) and tryptophan. In the presence of GQD-DMA, prepared by hydrothermal of GO-DMA, an electrostatic interaction formed through the coordination of Pb^2+^ with the carboxylate group of tryptophan and sulfur atoms on the surface of GQD-DMA, producing a rigid structure where Pb^2+^ acts as a cross-linker, resulting in the fluorescent enhancement of the system. They wrote that this was due to the strong energy-transfer interactions between tryptophan and DMA-GQDs. The system showed selectivity toward Pb^2+^ with a linear range of 0.01 nM to 1 nM, and the limit of detection was 0.009 nM, which was the lowest reported so far [69].

A new GQDs and L-cysteine (L-Cys) coreactant ECL system for sensing Pb^2+^ was developed by Dong et al. (2014). The GQDs were first obtained by the chemical oxidation of carbon black. They wrote that the oxidation of L-Cys, the presence of dissolved oxygen, and the reduction of GQDs contributed to the ECL signal. Pb^2+^ inhibited the formation of three kinds of free radicals (RSO^•^, RSO_2_^•^, and RSO_3_^•^) from the oxidation of L-Cys and thus quenching the ECL signal. A good linear relationship between the quenching ratio and concentration of Pb^2+^ was obtained in the range of 0.10 µM to 10 µM and the system can detect Pb^2+^ as low as 70 nM [70].

Subsequently, Pb^2+^ had also been detected using fluorescence nanosensors based on a GQDs–aptamer conjugate and GO where GQDs act as a fluorophore, while GO acts as an electron acceptor and quenching agent. Both GQDs and GO were obtained through the oxidation of graphite powder, while the further reduction of GQDs with excessive sodium borohydride produced rGQDs. After the fabrication of GQDs, it was assembled on the surface of GO through π–π stacking and electrostatic attraction, thus quenching the fluorescence. Upon the addition of Pb^2+^ into the aptamer–rGQDs probe, the fluorescence was quickly recovered where it was triggered by the complexation of aptamer-rGQDs/Pb^2+^. This work by Qian et al. in 2015 obtained a linear relationship between fluorescence intensity and Pb^2+^ concentration within a wide range of 9.9 nM to 435 nM, and the calculated limit of detection was 0.6 nM [71].

Later in 2016, Bian et al. prepared a yellow-emitting sulfur-doped GQDs (S-GQDs) via the hydrothermal process of 1,3,6-trinitropyrene, Na_2_S, and NaOH, which was then served as a fluorescent probe for Pb^2+^ detection. Pb^2+^ addition showed remarkable fluorescence quenched of S-GQDs compared to other metal ions, which was caused by a higher binding affinity with S and O functional groups of S-GQDs. The fluorescence intensity ratio and Pb^2+^ concentration in the range of 0.1 µM to 140 µM demonstrated a good linear relationship with a detection limit of 30 nM [72].

In 2018, Niu et al. combined GQDs with gold nanoparticles (AuNPs) through the pairing reaction between DNAs modified on both GQDs and AuNPs. As a result, fluorescence was easily quenched through fluorescence resonance energy transfer (FRET) between GQDs and AuNPs. Fluorescence recovery could be achieved upon the addition of Pb^2+^, where Pb^2+^ activates the catalytic strand and cleaves the linker DNAs to separate GQDs and AuNPs, leading to the detection of Pb^2+^. The proposed sensor showed a broad linear range of Pb^2+^ between 0.05–4 µM with a detection limit of 16.7 nM [73].

Another work by Sun et al. (2018) also detected Pb^2+^-based FRET in self-assembled multilayers. However, this time they used G-rich DNA as a linker to bridge GO and glutathione-functionalized GQDs (GQDs@GSH). The GQDs@GSH used in this work was prepared through the pyrolysis of citric acid and glutathione. The presence of a certain amount of Pb^2+^ induced the formation of the G-quadruplex, which led to a difference in chain length and shortening distance between GO and GQDs@GSH. As a result, the energy transfer was enhanced and the fluorescence of GQDs@GSH was quenched by GO within a Pb^2+^ concentration ranging from 2.4 nM to 11.5 nM. The value measured for the detection limit was 2.2 nM [74].

At the same time, Xu et al. (2018) innovatively prepared N, P, S co-doped GQDs (NPS-GQDs) using anthracite coal as raw material. The oxidation of the coal in HNO_3_ and H_2_SO_4_ eventually produced NPS-GQDs. Then, it was used as a fluorescent probe for Pb^2+^ detection. Due to the strong binding affinity and fast chelating kinetics of Pb^2+^ with the carboxyl and sulfhydryl groups of NPS-GQDs, the fluorescence of NPS-GQDs was effectively quenched through the electron transfer process within GQDs. A good linear correlation between fluorescence intensity and Pb^2+^ concentration that ranged within 1 µM to 10 µM was observed with a limit of detection of 750 nM [75].

The latest work by Kaewprom et al. (2019) synthesized diethyl dithiocarbamate-doped GQDs (DDTC-GQDs) through the pyrolysis of citric acid and DDTC. It was further applied as a resonance light scattering (RLS) probe for the determination of Pb^2+^. The RLS intensity of DDTC-GQDs was linearly increased in the presence of Pb^2+^ within a concentration of 4.83 nM to 48.3 nM, resulting from the enhancement mechanism. In short, the interaction between Pb^2+^ and DDTC-GQDs led to the formation of a stable and bigger volume of the complexes. The detection limit was calculated to be 3.86 nM [76]. Table 4 shows the findings of different incorporations of GQDs with an optical sensor toward lead ion detection.

### 2.4. Copper Ion (Cu^2+^)

Copper is an essential element for all living organisms, including maintaining the metabolism of the human body. At a high dose, it can cause eczema, stomach ache, intestinal irritation, and liver and kidney damage, while the deficiency of Cu^2+^ leads to mental retardation, anemia, and hypothermia [77,78]. Therefore, the daily intake of Cu^2+^ needs to be controlled to avoid more critical problems from happening. These are the updated studies on the GQDs-based optical sensing of Cu^2+^.

Sun et al. managed to achieve 6.9 nM as the lowest detection limit for the introductory work of Cu^2+^ detection using a GQDs-based optical sensor in 2013. First, microwave irradiation was conducted to prepare gGQDs from GO. Then, they fabricated amino-functionalized gGQDs (afGQDs) by the hydrothermal amination of GQDs before using it as a fluorescent probe to detect Cu^2+^. Their success was based on the binding affinity of Cu^2+^ ion with N and O on the surface of afGQDs being higher than that of the other transition metal ions. The surface charge of GQDs converted to positive, which also made the cellular uptake of GQDs toward Cu^2+^ easier. The fluorescence intensity was proportional to the increase in concentration of Cu^2+^ up to 100 nM [79].

There were a lot of related studies in the following year. Wang et al. (2014) synthesized GQDs via the hydrothermal method from re-oxidized GO. Then, the prepared GQDs were used as a fluorescent probe to detect Cu^2+^ efficiently. Over the range of 0 to 15 µM, the fluorescence intensity of GQDs decreased, with increasing Cu^2+^ concentration resulting from the static mechanism of the complexation of Cu^2+^ by GQDs. They also obtained the limit of detection value of 226 nM [80].

In 2014, Liu et al. proposed an optical sensor based on GQDs for the detection of Cu^2+^ after realizing that most of the previous materials that have been used to detect Cu^2+^ released toxic metal ions. Therefore, they synthesized the GQDs by the chemical oxidation of pitch graphite fibers and used it as a platform for Cu^2+^ sensing. Most of the metal ions, including Cu^2+^, “turn off” the PL intensity of GQDs. However, after adding biothiol cysteine, only Cu^2+^ “turns on” the PL. A linear relationship was observed for PL intensity against concentrations of Cu^2+^ over the range of 0 to 0.20 mM with a detection limit of 330 nM [81].

A year later, Liu and Kim synthesized GQDs from a carbon nanoonion precursor via chemical oxidation and dialyzed them, forming two parts: outside (emit UV emission) and inside (emit blue PL) the dialysis bag. Then, GQD-B and GQD-UV were compared for use as sensors to detect heavy metal ions. The GQD-B and GQD-UV were sensitive toward Cu^2+^ and Fe^3+^ respectively, which was probably due to the difference in the binding affinity of the GQDs with the metal ions. For the optical detection of Cu^2+^ using GQD-B, the PL intensity decreases with an increasing concentration of Cu^2+^ between 20–200 nM where the detection limit was found to be 20 nM [82].

Another study by Lin et al. (2015) showed that europium-decorated GQDs (Eu-GQDs) prepared via the strong acid treatment of 3D Eu-graphene successfully changed the electron density and chemical activities on the GQDs’ surface. On top of that, it was used as a fluorescent probe for the detection of Cu^2+^. The coordination reaction between Cu^2+^ and carboxyl groups on the surface of Eu-GQDs quenched the fluorescence intensity of Eu-GQDs. Compared with other metal ions, Cu^2+^ showed a significant linear relationship in the range of 0.1 µM to 10 µM with a detection limit of 56 nM [83].

At the same time, a dual-photoluminescence probe for Cu^2+^ was presented by Sun et al. (2015). The probe consisted of blue light-emitting glutathione-functionalized GQDs (GQDs@GSH) as the internal standard and yellow light-emitting CdTe QDs as the sensing fluorophore. The GQDs@GSH used in this work was obtained through a one-step pyrolysis of citric acid and glutathione. Due to the addition of Cu^2+^, the PL color of blue GQDs@GSH remained unchanged. However, when CdTe QDs was mixed with GQDs@GSH, the color changed from yellow to pink, purple, and blue. The ratio of the PL intensity also increased with the concentration of Cu^2+^ within the range of 0.1 µM to 1.0 µM, and the calculated limit of detection was 53 nM [84].

In the following year, Li et al. (2016) innovatively enriched the surface of GQDs, which was prepared using the electrochemical cyclic voltammetry technology of graphene film with a carboxyl group through a universal, mild, and in situ post-treatment method. The proposed method also helps the Cu^2+^-T-GQDs complexation to occur. The treated GQDs, which were named T-GQDs, were synthesized via the post-oxidation approach of GQDs in potassium persulfate (K_2_S_2_O_8_) and used as a fluorescent probe for the detection of Cu^2+^. A good linear relationship was obtained between the concentration of Cu^2+^ and the fluorescence intensity of T-GQDs in the range of 0 to 20 µM. The T-GQDs can also detect Cu^2+^ as low as 20 nM [85].

In 2018, Wang et al. demonstrated a sensitive and selective sensing of Cu^2+^ in a rat brain for the first time using GQDs as a fluorescent nanosensor. Firstly, they synthesized the GQDs through the chemical oxidation of three-dimensional (3D) nanomesh graphene frameworks before using it as a nanosensing platform. The formation of complexation between Cu^2+^ and GQDs produced a static quenching that quenched the fluorescence of GQDs. The fluorescence ratio showed a good linear relationship with Cu^2+^ concentration within 0.1 µM to 1.0 µM, and the limit of detection was calculated to be 67 nM [86]. Table 5 covers all of the optical sensing of copper ions using GQDs.

### 2.5. Silver Ion (Ag^+^)

Nowadays, silver-based products have been extensively used in our lives, and in fact, tonnes of silver have been released to the environment from industrial waste. Therefore, it is important to test the toxicity of silver and its derivatives. A study showed that Ag^+^ has a higher toxicity compared to other derivatives of silver [87]. Ag^+^ has also been assigned as one of the most toxic forms of heavy metal ions in the same class as Cd^2+^, Cu^2+^, and Hg^2+^, which can cause irritation of the eyes, skin, respiratory, and intestinal tract, and changes in blood cells [88]. Due to these reasons, it has drawn researchers’ attention to detect Ag^+^, especially using GQDs-based optical sensors.

The earliest work of Ag^+^ detection using a GQDs-based material was reported by Ran et al. (2013). A label-free, rapid, and ultrasensitive method for the detection of Ag^+^ and biothiol was conducted based on the as-prepared Ag nanoparticle-decorated GQDs (AgNPs/GQDs). GQDs were prepared using the same method as in [89]. Briefly, it was stated that the formation of Ag nanoparticles on GQDs will produce quenching effects on QDs, which helped to detect Ag^+^. It was also found that the fluorescence intensity was linearly proportional to the Ag^+^ concentration up to 100 nM, and they were able to get the lowest limit of detection with a value of 3.5 nM [90].

A year later, a work on GQDs synthesized from biowaste (dead leaves) by Suryawanshi et al. (2014) was published. Carbon powder obtained from the dead leaves can be turned into GQDs through a hydrothermal reaction. Then, the GQDs were further modified to produce amine-terminated GQDs (Am-GQDs) with higher PL intensity and dispersibility than GQDs, which then were used as a fluorescent probe and surprisingly have high selectivity toward Ag^+^. The fluorescence quenching (switch-off) was observed by Ag^+^ and some metal ions; however, only Ag^+^ switched-on the fluorescence back with the addition of cysteine. The obtained value for the limit of detection was about 300,000 nM [91].

A fluorescent probe for Ag^+^ detection in aqueous solution was also built by Tabaraki and Nateghi in 2016 based on nitrogen-doped GQDs (N-GQDs). The N-GQDs were prepared via a microwave-assisted hydrothermal reaction of glucose and ammonia. They stated that the fluorescence of N-GQDs was notably quenched in the presence of Ag^+^, which was due to the charge transfer process. This process happened when the distance of Ag^+^ and N-GQDs was sufficiently short for the charge to transfer, resulting in the quenching of PL. The linear range of N-GQDs was estimated to be 0.2 µM to 40 µM with a calculated value of 168 nM as the limit of detection [92].

Due to the awareness of the effects of long-term exposure to Ag^+^, Bian et al. (2017) synthesized sulfur-doped GQDs (S-GQDs) from 1,3,6-trinitropyrene through a hydrothermal method that extremely improved the surface chemical reactivity of GQDs. The fluorescence quenching exhibited by S-GQDs in the work was suitable to be used as sensing probes for the determination of Ag^+^ ions, which were produced a wide linear range of 0.1 µM to 130.0 µM, and the limit of detection of 30 nM was calculated [93].

A work by Kaewanan et al. (2017) used GQDs prepared through citric acid pyrolysis for the determination of Ag^+^. Briefly, Hg^2+^ was first bounded with GQDs; upon the addition of hydrolyzed thioacetamide (TAA), TAA reacted with Hg^2+^ through Hg–S interaction until the Hg^2+^–GQDs complex dissociated, resulting in the fluorescence being restored. Afterwards, when Ag^+^ was added, the intensity of GQDs decreased. The fluorescence turn-off system of GQDs decorated with Hg^2+^ and TAA produced a linear range between 0.5 µM–10.0 µM and limit of detection value of 180 nM under optimized conditions [94].

Inspired by a recent study conducted by Yang and Wang [95], Zhao et al. (2017) reported a ratiometric fluorescent sensor based on GQDs as a reference fluorophore and o-phenlyenediamine (OPD) as specific recognition probes for the detection of Ag^+^. In short, the Ag^+^ ions oxidized OPD to form DAP with increasing fluorescence intensity and simultaneously, the fluorescence intensity of GQDs was quenched by generating the DAP through FRET. This group also obtained a linear range of up to 115.2 µM with a detection limit of 250 nM [96]. The optical sensor findings based on GQDs for silver ion detection are tabulated in Table 6.

### 2.6. Other Toxic Metal Ions

Other naturally occurring metal ions that also have high toxicity include aluminum ion (Al^3+^), cadmium ion (Cd^2+^), cobalt ion (Co^2+^), and nickel ion (Ni^2+^). Heavy metal-contaminated wastes usually contain more than one heavy metal ion, including all of the ions that have been mentioned before. These toxic metal ions sometimes can interfere during the main process that happens in the human body, which then leads to excessive damage to the body without proper measures taken [97]. Optical sensing of these toxic metal ions using GQDs-based materials may help to combat metal ions pollution.

An introductory work on the optical detection of Al^3+^ using a GQDs-based material was proposed and conducted by Fan et al. in 2014, where they demonstrated the boron-doping graphene quantum dots (B-GQDs) synthesized through an electrochemical approach of the graphite rod. Due to the strong fluorescence properties displayed by B-GQDs, they used it in fluorescent chemosensors for Al^3+^ detection. The fluorescence intensity was extremely enhanced with increasing Al^3+^ concentration up to 100 µM. A detection limit value of 3660 nM was also obtained [98].

There were no other studies using GQDs-based materials for Al^3+^ detection until 2018, when Fang et al. prepared nitrogen-doped GQDs (N-GQDs) through the solvothermal process of GO using dimethylformamide and used it as a fluorescent probe to detect Al^3+^. This is based on the knowledge that N-GQDs will undergo a photo-induced electron transfer (PET) process resulting from the formation of complexation between Al^3+^ and N-GQDs. Eventually, the fluorescence intensity of N-GQDs remarkably increased with the increment of Al^3+^ concentration in the range of 2.5 µM to 75 µM, and the limit of detection was found to be 1300 nM [99].

In 2012, GQDs were initially used for Cd^2+^ sensing in the work by Li et al. They prepared stabilizer-free greenish yellow-luminescent GQDs (gGQDs) from GO nanosheets using the microwave-assisted method under acidic conditions, and based on its intense electrochemiluminescence (ECL), a novel ECL sensor for Cd^2+^ was proposed. This sensor worked based on the competitive coordination for metal ions between cysteine as a chelator with GQDs. A linear relationship was observed between Cd^2+^ concentration with ECL intensity, which decreased over the range of 20 nM to 150 nM with a detection limit of 13 nM [89].

Zhang et al. (2015) also reported the synthesis of nitrogen-doped GQDs (NGQDs) through the hydrothermal oxidize method of nitrogen-doped graphene. They wrote that the synthesized NGQDs (auxiliary coordination agents) help to accelerate the coordination reaction of Cd^2+^ with 5,10,15,20-tetrakis(1-methyl-4-pyridinio) porphyrin tetra(p-toluenesulfonate) (TMPyP). The presence of Cd^2+^ in the range of 0.5 µM to 8 µM decreased the fluorescence intensity of the probe, while the detection limit was found to be 88 nM [100].

So far, there was only one work done for the optical detection of Co^2+^ based on GQDs in 2016. Chen et al. developed a dual-potential ratiometric responsive chemiluminescence (ECL) sensor based on nitrogen-doped GQDs (NGQDs), which were prepared via the reduction of nitrogen-doped GO. They claimed that the enhancement of ECL intensity was due to the catalytic action played by Co^2+^ on the intermediate species in ECL reactions. Thus, the ratio of ECL intensities at two excitation potentials was used to measure the target, Co^2+^. They found that the intensity change of ECL was linearly dependent on the square of Co^2+^ concentration in the range of 1.0 µM to 70 µM. The estimated limit of detection was 300 nM [101].

The only work for the optical detection of Ni^2+^ based on GQDs was proposed by Huang et al. Back in 2013, they revealed that GQDs can be used to detect Ni^2+^ based on a quenching-recovery strategy with the help of dimethylglyoxime (DMG) as the recovery agent. The GQDs were achieved by the chemical oxidation of carbon fibers. Briefly, photoinduced electron transfer from GQDs to a metal ion with partial d orbits led to the perturbation and non-radiative transitions of GQDs, resulting in PL quenching. Upon the introduction of DMG as a chelator, the interaction of GQDs with Ni^2+^ was weakened, causing PL recovery. A linear range of up to 90 µM and the detection limit of 4100 nM were also obtained [102]. Table 7 summarizes and compares the incorporation of GQDs in various optical methods for the detection of other toxic metal ions such as Al^3+^, Cd^2+^, Co^2+^, and Ni^2+^.

## 3. Emergence of Surface Plasmon Resonance as Alternative Optical Sensors for Metal Ion Detection

Surface plasmon (SP) is a phenomenon where free electrons on the surface of a metal–dielectric interface collectively oscillate when interacting with incident electromagnetic waves. The momentum of the SP can be described by a vector function with both magnitude and direction. Resonance occurs when the SP is coupled with the incident electromagnetic wave, hence the term surface plasmon resonance (SPR). SPR is very sensitive to the refractive index immediately adjacent to the thin film. Over the past years, SPR has become one of the well-known emerging optical methods developed for metal ion sensing. In order to generate SPR, there are a few approaches to provide coupling between the SP and electromagnetic wave, which includes grating coupling and prism coupling SPR.

**Figure 2:**
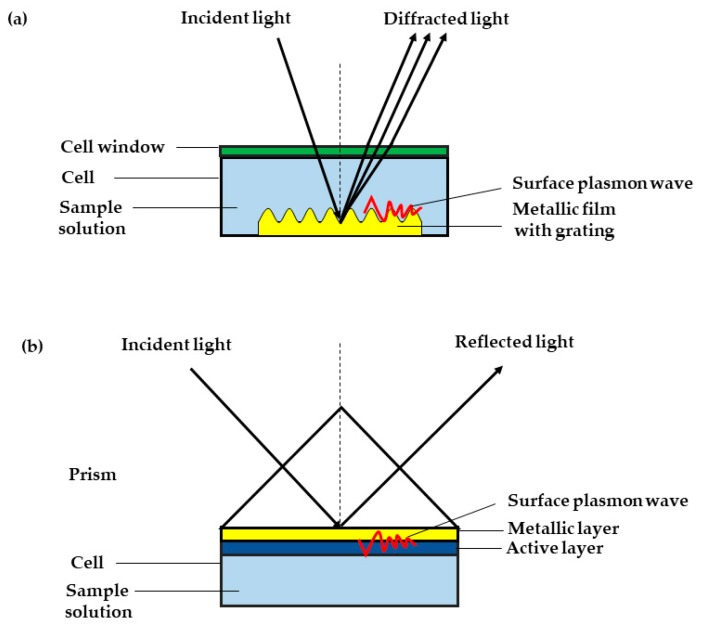
Surface plasmon resonance (**a**) grating-based and (**b**) prism-based (Kretschmann configuration).

The excitation of SP by the grating-based SPR phenomenon was first presented by Wood in 1902 [103]. In this method, a light wave is incident from a dielectric medium to metal grating to generate SP, as shown in Figure 2a. When the light wave is made incident on the surface of the grating, diffraction gives rise to a series of diffracted waves, which was directed away from the surface at a variety of angles. The diffracted wave can couple with the SP if the momentum of the diffracted wave is equal to the SP [104]. Grating-based SPR has been demonstrated to use light intensity and wavelength interrogation for data acquisition [105,106]. Grating-based SPR has many advantages; for example, it offers much higher miniaturization and integration capabilities [107]. However, grating-based SPR is less often adopted in sensing applications, as it has lower sensitivity compared to the prism-based SPR [108,109]. Another drawback of grating-based SPR is the mathematics involved in modeling the SPR structures, as these are more complicated than the prism-based SPR, which in turn causes the data analysis to be more difficult [110,111]. On the other hand, prism-based SPR is the most widely used in commercial SPR devices due to its high sensitivity and ease of use [112,113]. Prism-based SPR can be divided into two configurations, i.e., the Kretschmann configuration and Otto configuration.

In 1960s, Kretschmann and Otto demonstrated the optical excitation of surface plasmons by means of attenuated total reflection. Since the first demonstration of SPR for the learning of processes happened at the surface of metal and sensing of gases, SPR has been intensively studied and vast advances have been made in the development of technology and its application [114]. The Kretschmann configuration is normally used in most SPR applications, where a metal such as gold, silver, copper, and aluminum, all of which carry a large number of free electrons, is placed at the interface of two dielectric media. Since gold is the most stable and sensitive, it is favorably used as the metal film. When plane-polarized light hits the gold-coated film prism under total internal reflection conditions, SPR will eventually occur. Then, the reflected beam will be detected for processing. The Kretschmann configuration diagram is shown in Figure 2b. The SPR method enables researchers to study the interaction between immobilized receptors and analytes in solution, in real time, and without labeling the analytes. Furthermore, SPR also provides information on the specificity, kinetics, and affinity of the interaction, or the concentration of the analytes by observing the binding rates and binding levels [115].

The most important finding in the SPR sensor is the active layer development. The active layer is placed in between the metal layer and the cell. Starting from 2001, incorporating a metal film with an active layer of materials such as semiconductors, biopolymers, conducting polymers, dyes, and many more have been proven to improve the sensitivity of SPR sensor for metal ions detection [116,117,118,119,120,121,122,123,124,125,126,127,128,129,130]. Up until 2019, there was no study on the incorporation of SPR with GQDs-based material. However, graphene and its derivatives are believed to improve the performance and sensitivity of the SPR sensor toward metal ions, since they have the ability to immobilize other nanoparticles [131]. Subsequently, there were many reports on the SPR sensor incorporated with graphene-based materials such as graphene oxide (GO) for the detection of toxic metal ions.

The use of GO as active layer has stepped up the game in SPR sensing. The change of the atomic structure from graphene to GO made it the best candidate to incorporate with SPR, since it acts as a precise transport medium for the selective permeation of ions [132]. An introductory work was done by Lokman et al. in 2014 where this group successfully enhanced the sensitivity of the SPR sensor by developing a gold-chitosan-graphene oxide (Au/CS/GO) nanostructured thin film for Pb^2+^ detection. The Au/CS/GO thin film was compared with the Au/CS thin film in all aspects, including SPR response. By incorporating GO to Au/CS, they observed a rougher surface, which they believed could improve the adsorption of Pb^2+^ onto Au/CS/GO thin film. Upon the exposure of both thin films to different concentrations of Pb^2+^, it can be seen that both thin films sensitively detect Pb^2+^ as low as 0.03 ppm, besides having a good linear relationship ranging from 0.03 ppm to 5 ppm. Excitingly, the Au/CS/GO thin film also showed noticeable changes in incidence angle compared to the Au/CS thin film, and thus has a higher potential to measure a wide range of Pb^2+^ values. Furthermore, the Au/CS/GO thin film also showed higher sensitivity than the Au/CS thin film with the values of 1.11200° ppm^−1^ and 0.77600° ppm^−1^, respectively [133].

A work by Kamaruddin et al. (2016) improved the performance of the chitosan–graphene oxide (CS–GO) SPR sensor by implementing a multimetallic layer of Au–Ag–Au nanostructure to the CS–GO layer. They also used the obtained sensor to detect Pb^2+^. The shift of SPR angle increased up to 3.5° using the proposed structure compared to a single gold layer of CS–GO (Au–CS–GO) as a result of the enhanced evanescent field at the sensing layer–analyte interface. They observed a great increase in value for the shift of SPR angle using Au–Ag–Au CS–GO from 0.1 ppm to 1 ppm of Pb^2+^. However, the gradual shift of SPR angle observed during 3 ppm to 5 ppm could be due to the saturation of active binding sites on the CS–GO layer. The calculated sensitivity of the developed sensor was 2.05° ppm^−1^ and 0.29° ppm^−1^ for 0.1 to 1 ppm and 1 ppm to 5 ppm, respectively. The Au–Ag–Au CS–GO SPR sensor can detect Pb^2+^ as low as 0.1 ppm [134].

A year later, Kamaruddin et al. (2017) once again developed a SPR sensor with the same layer (Au/Ag/Au/CS–GO). The only difference this time was that they used the sensor to detect Pb^2+^ and Hg^2+^ with the concentration ranging from 0.1 ppm to 5 ppm and compared their performance, especially the binding affinity between the metal ions and CS–GO sensor. They observed that Au/Ag/Au/CS-GO has a higher sensitivity toward Pb^2+^ with the same values as the previous study, 2.05° ppm^−1^ compared to Hg^2+^ with a lower sensitivity of 1.66° ppm^−1^. Then, they calculated the binding affinity constant for both metal ions, which was found to be 7 × 10^5^ M^−1^ for Pb^2+^ and 4 × 10^5^ M^−1^ for Hg^2+^, signifying that the CS–GO sensing layer was more favorable to Pb^2+^. They wrote that this was due to the greater electronegativity and ionic radii of Pb^2+^ [135].

The detection of Co^2+^ has also been conducted by Saleviter et al. in 2017. They used immobilized 4-(2-pyridylazo) resorcinol in a chitosan–graphene oxide composite (PAR-Cs-GO) as the active layer. They highlighted that coating PAR-Cs-GO on top of a gold layer caused a shift in the resonance angle to the right. The prepared sensor has a sensitivity value of 0.00069°ppm^−1^ and is able to detect Co^2+^ as low as 10 ppb [136]. Another comparative study of Co^2+^ detection done by the same author, Saleviter et al. (2018), has also been done. However, currently, they used a different active layer, which was a cadmium sulfide quantum dot–graphene oxide–chitosan (CdS QDs-GO-Cs) nanocomposite thin film. For the sensing part, it was stated that the CdS QDs-GO-Cs changed the refractive index of the active layer, resulting in a shift in the resonance angle. [137]

Another work by Zainudin et al. (2018) reported the incorporation of a novel valinomycin-doped chitosan–graphene oxide (CS-GO-V) thin film with SPR for potassium ion (K^+^) sensing. Basically, the CS-GO-V was deposited on top of the gold surface using the spin coating technique. Then, the bare gold thin film and CS-GO-V thin film were used to observe the SPR response for K^+^ in solution. When exposing the gold thin film to K^+^, the resonance angle did not show any shifting for all of the concentrations ranging from 10 to 100 ppm. However, when replacing with a gold/C-GO-V thin film, the resonance angle shifted to the right. As the concentration of K^+^ increases, the resonance angle also increased, which can be attributed to the immobilization of valinomycin, which has a strong affinity toward K^+^. The system has a high sensitivity value of about 0.00948° ppm^−1^ with a detection limit of 0.001 ppm (25.57 nM) [138].

In a recent work by Daniyal et al. (2018), nanocrystalline cellulose modified by hexadecyltrimethylammonium bromide and GO composite (CTA-NCC/GO) has been used as an active layer. It was coated on top of a gold thin film using the same technique as Zainudin et al. [138]. Then, this layer was used to detect Cu^2+^. They obtained a high sensitivity of 3.271° ppm^−1^ for a low concentration of Cu^2+^ ranging from 0.01 ppm to 0.1 ppm. The sensing performance of the SPR sensor toward Cu^2+^ was enhanced with the presence of CTA-NCC/GO compared with a bare gold thin film, and surprisingly, they obtained a high binding affinity constant of 4.075 × 10^3^ M^−1^ [139]. Likewise, Daniyal et al. once again incorporated CTA-NCC/GO with an SPR sensor to detect Ni^2+^. Compared with a bare gold thin film, CTA-NCC/GO showed good performance when detecting Cu^2+^. From 0.01 ppm to 0.1 ppm of Ni^2+^ concentration, the calculated sensitivity of the sensor was 1.509° ppm^−1^ with a binding affinity constant of 1.620 × 10^3^ M^−1^ [140].

## 4. Future Trends in the Development of Graphene Quantum Dots-Based Surface Plasmon Resonance Optical Sensor for Toxic Metal Ion Detection

Up until now, metal ions contamination has still been a serious problem. Beyond a specific limit, the exposure to metal ions can cause harm and affect human health severely as much as it affects the environment. For these reasons, researchers have to immediately find the solution to fight the problem. One of the efforts is obviously by an early detection of metal ions at a concentration below the danger limit. Therefore, tremendous efforts devoted to developing sensors with high sensitivity and low detection limits are much needed nowadays. SPR has many advantages such as being label-free and having high sensitivity, low cost, and fast response, which made it the best candidate to be used for the optical detection of metal ions [141]. Most scientists have competed to produce an SPR sensor with high sensitivity and selectivity toward metal ions by finding the best active layer coated on top of the gold film or modifying them, since it may influence the performance of the SPR sensor.

It is believed that the incorporation of a GQDs-based material with an SPR sensor has high potential to detect toxic metal ions [142]. Consequently, a preliminary work was done, where chitosan/carboxyl-functionalized GQDs (Au/Cs/CGQDs) were coated on top of the gold thin film and incorporated with SPR spectroscopy to detect Hg^2+^. Firstly, only a gold thin film followed by a Au/Cs/CGQDs thin film were tested to detect deionized water. As a result, the gold-only thin film had a lower resonance angle than Au/Cs/CGQDs. After the addition of Hg^2+^ with increasing concentrations from 10 ppm to 100 ppm, the change in resonance angle was directly increased. This positive result may be due to the interaction formed between Hg^2+^ and Cs/CGQDs, where the formation of a pair of electrons shared between Hg^2+^ with positive charge and amine-functional group in chitosan with negative charge occurred. The Au/Cs/CGQDs can detect Hg^2+^ from as low as 0.5 ppm (2490 nM) with a sensitivity of 0.00062° ppm^−1^ [143]. This work proved that GQDs are able to enhance the sensitivity of the built sensor.

Unfortunately, the study on the SPR sensor incorporated with a GQDs-based material for toxic metal ions detection remains inchoate. Therefore, it is of interest to enhance the sensitivity of the SPR sensor using a GQDs-based material by immobilizing it with other materials, which can increase the sensing performance of the sensor to its full potential. In view of the unique features of the GQDs-based material that have been mentioned before, it is expected to have good incorporation with SPR to detect toxic metal ions.

## 5. Conclusions

This paper reviewed the systematic and collective progression of optical sensors for toxic metal ions detection using GQDs-based materials since they were first introduced. Additionally, the usage of graphene-based materials incorporated with SPR sensor to detect metal ions has also been discussed and reviewed. With the superior properties of GQDs, it is envisioned that the application of GQDs-based materials in sensing applications will continue to expand. The incorporation of GQDs in the development of a novel and excellent SPR sensor is significant, because it will enormously improve the sensitivity and selectivity for toxic metal ions sensing with the lowest concentration as possible in the future.

## Figures and Tables

**Table 1:** The advantages and disadvantages of optical sensors. SPR: surface plasmon resonance.

Optical Sensors	Advantages	Disadvantages
Fluorescent	High sensitivity and selectivity; real-time measurement; good reproducibility	Slightly slow detection; time consuming; limited application (small molecules)
Electrochemiluminescence	Good sensitivity and selectivity; stable; strong anti-interference ability; wide detection range	High cost; low compatibility; complicated preparation; frequent electrode fouling
Photoluminescent	High sensitivity and selectivity; real-time measurement; good reproducibility	Low precision and accuracy; time consuming; limited application (small molecules)
Colorimetric	Good sensitivity; fast detection; inexpensive	Low reproducibility; low stability; low selectivity
SPR	Very high sensitivity; simple; low cost; label-free	Low selectivity (improving)

**Table 2:** The GQDs-based optical sensor for ferric ion detection.

Type of GQDs	Synthesis Method	Starting Materials	Optical Method	Linear Range	LOD ^1^ (nM)	References
P-GQDs	carbonization/hydrothermal	pyrene/hydrazine hydrate	Fluorescent probe	-	5	[22]
N-GQDs	pyrolysis/hydrothermal	citric acid/hydrazine	Fluorescent probe	1–1945 µM	90	[23]
BMIM^+^-GQDs	electrochemical cutting	3D graphene	Fluorescent sensor	0–80 µM	7220	[19]
N-GQDs	acid vapor cutting	MOF-derived carbon	Fluorescent probe	1–70 µM	80	[24]
N-GQDs	carbonization/hydrothermal	citric acid/ammonia	Fluorescent probe	1–500 µM	1000	[25]
S-GQDs	electrolysis	graphite/sodium p-toluensulfonate	Fluorescent probe	0–0.7 µM	4.2	[26]
NA-GQDs	thermolysis	glycine	Fluorescent sensor	0.5–500 µM	100	[27]
GQDs	acid vapor cutting	SiO_2_(SBA-15)	Fluorescent probe	3–60 µM	300	[28]
GQDs	pyrolysis	citric acid	Dual-channel fluorescent probe	10–200 µM	10 000	[29]
RBD-GQDs	electrochemical exfoliation/acidic oxidation	graphite rod/rhodamine B	Fluorescent sensor	0–1 µM	20	[30]
GQDs	microwave/pyrolysis	aspartic acid/NH_4_HCO_3_	Fluorescent probe	0–50 µM	260	[31]
SL/GQDs	pyrolysis/hydrothermal	citric acid/SL/NaOH	Fluorescent sensor	0.005–500 µM	0.5	[32]
GQDs/PS-AER	acid oxidation/absorption	graphite/PS-AER	Fluorescent sensor	1–7 µM	650	[33]
DA-GQDs	pyrolysis/covalent conjugation	citric acid/dopamine	Fluorescent probe	0.02–2 µM	7.6	[34]
AL-GQDs	Solution chemistry/amidation	GO/amino acid	Fluorescent probe	0.05–200 µM	50	[35]
DPA-GQDs	pyrolysis	citric acid/D-penicillamine	Fluorescent probe	0.004–1.8 mM	1200	[36]
BGQDs	electrolysis	graphite rod/borax solution	Fluorescent probe	0.01–100 µM	5	[21]
N-GQDs	hydrothermal	glutathione/Ag+	Fluorescent probe	50–2000 µM	70	[37]
N, S-GQDs	hydrothermal	1,3,6-trinitropyrene/thiourea/DMF/sodium hydroxide	Fluorescent probe	0.01–25.0 µM	8	[38]
N, S-GQDs	dehydration	citric acid/L-cysteine	Fluorescent probe	0.01–3 μM	3.3	[39]
GQDs	oxidation	carbon black/nitric acid	Fluorescent probe	0–60 μM	450	[40]
RH-GQDs	hydrothermal	rice husk	Fluorescent sensor	0–1 mM	5.8	[41]
N, Fe-GQDs	hydrothermal	ammonium iron (III) citrate	Fluorometric and Colorimetric dual-mode sensor	10–110 μM 0–450 μM	3210 1340	[42]
af-GQDs	chemical oxidation/hydrothermal	high-softening point asphalt/ammonia	Fluorescent probe	0–50 µM	0.51	[43]

^1^ where LOD is limit of detection. af: amino-functionalized, AL: l-analine, B: boron, DA: dopamine, DMF: dimethylformamide, DPA: D-penicillamine, GO: graphene oxide, MOF: metal-organic framework, N: nitrogen, NA: nitrogen and amino acid, P: photoluminescent, PS-AER: polystyrenic anion-exchange resin, RBD: rhodamine B derivative, RH: rice husk, S: sulfur, SL: ligninsulfonate.

**Table 3:** The GQDs-based optical sensor for mercury ion detection.

Type of GQDs	Synthesis Method	Starting Materials	Optical Method	Linear Range	LOD ^1^ (nM)	References
GQDs	carbonization	citric acid	Fluorescent chemosensor	–	3360	[46]
GQDs	ultrasonic route	graphene	Fluorescent probe	0.8–9 µM	100	[47]
GQDs	pyrolysis	citric acid	Dual fluorescent sensor	1–50 nM	0.439	[48]
DNA-GQDs	hydrothermal cutting	graphite powder	Fluorescent probe	0.001–10 µM	0.25	[49]
N-OGQDs	microwave-assisted hydrothermal	citric acid/L-DOPA	Fluorescent probe	0.04–6 µM	8.6	[50]
GQDs-SR	Hummers method	graphite powder/SR	Fluorescent chemsensor	0.6–12 µM	230	[51]
CdTe@SiO_2_@GQDs	pyrolysis	citric acid	Ratiometric fluorescent probe	0.01–22 µM	3.3	[52]
cys-GQDs	carbonization	citric acid/cysteine	Fluorescent probe	0–500 µM	20	[53]
GQDs-T-ZnPc	Hummers method	GO/T-ZnPc	Fluorescent “turn ON”	0.1–20 nM	0.05	[54]
Val-GQDs	pyrolysis	citric acid/valine	Fluorescent probe	0.8–1000 nM	0.4	[55]
GQDs-Pcs	hydrothermal	graphite powder	Fluorescent “turn ON”	0.5–50 nM	0.12	[56]
Fe_3_O_4_@SiO_2_@GQDs	pyrolysis	citric acid/Fe_3_O_4_@SiO_2_	Fluorescence detection	0.1–70 µM	30	[57]
PEI-GQDs	Hummers method	graphite powder/PEI	Fluorescent “off-on-off”	0.5–25.0 nM	0.25	[58]
MEA-GQDs	pyrolysis/functionalization	citric acid/MEA	Fluorescent “off-on”	0.05–5 µM	10	[59]
N, S-GQDs	hydrothermal	citric acid/thiourea	Fluorescent probe	0.1–15 µM	0.14	[60]
Mn(II)-NGQDs	hydrothermal	glycine/Mn^2+^/sodium citrate	Fluorescent probe	0–3.5 µM	0.34	[61]
PEHA-GQD-DPA	pyrolysis	citric acid/DPA/PEHA	Fluorescent probe	0.1–200 µM	0.046	[62]
N-GQDs	hydrothermal	citric acid/ammonia	Fluorescent probe	0.02–1 µM	4.7	[63]
N, S/GQDs	pyrolysis	citric acid/D-penicillamine	Fluorescent probe	0.9–30 nM	0.69	[64]
RhB-GQDs	hydrothermal	citric acid/rhodamine B/ethylenediamie	Fluorescent probe	0–10 nM	0.16	[65]
GQDs-DNA-AuNP	pyrolysis	citric acid/DNA/AuNP	ECL sensor	0.01–100 nM	0.00248	[66]

^1^ where LOD is limit of detection. AuNP: Au nanoparticle, DPA: D-penicillamine, ECL: electrochemiluminescence, GO: graphene oxide, L-DOPA: 3,4-dihydroxy-L-phenylalanine, MEA: monoethanolamine, Mn(II)-N: (Mn^2+^)-bonded nitrogen, N: nitrogen, Pcs: phthalocyanines, PEHA: pentaethyleneheaximine, PEI: polyethyleneimine, RhB: rhodamine B, S: sulfur, SR: spirolactam rhodamine, T-ZnPc: thymine-appended zinc phtalocyanine.

**Table 4:** The GQDs-based optical sensor for lead ion detection.

Type of GQDs	Synthesis Method	Starting Materials	Optical Method	Linear Range	LOD ^1^ (nM)	References
GQD-DMA	hydrothermal	GO-DMA	Fluorescent probe	0.01–1 nM	0.009	[69]
GQDs/L-Cys	chemical oxidation	carbon black	ECL	100–1000 nM	70	[70]
rGQDs	oxidation/reduction	graphite powder	Fluorescence “turn ON”	9.9–435 nM	0.6	[71]
S-GQDs	hydrothermal	pyrene/1,3,6-trinitropyrene	Fluorescent probe	0.1–140.0 µM	30	[72]
GQDs and AuNPs	purchased	-	FRET	0.05–4 µM	16.7	[73]
GQDs@GSH	pyrolysis	citric acid/glutathione	FRET	2.4–11.5 nM	2.2	[74]
NPS-GQDs	electrochemical oxidation	anthracite coal	Fluorescent probe	1–20 µM	750	[75]
DDTC-GQDs	pyrolysis	citric acid/DDTC	RLS	4.83–48.3 nM	3.86	[76]

^1^ where LOD is limit of detection. AuNPs: Au nanoparticles, DDTC: diethyl dithiocarbamate, DMA: 3,9-dithia-6-monoazaundecane, FRET: fluorescence resonance energy transfer, GO: graphene oxide, GSH: glutathione, L-Cys: L-cysteine, NPS- nitrogen, phosphorus and sulfur, RLS: resonance light scattering, S: sulfur.

**Table 5:** The GQDs-based optical sensor for copper ion detection.

Type of GQDs	Synthesis Method	Starting Materials	Optical Method	Linear Range	LOD ^1^ (nM)	References
afGQDs	microwave/hydrothermal amination	GO	Fluorescent probe	0–100 nM	6.9	[79]
GQDs	hydrothermal	reoxidized GO	Fluorescent probe	0–15 µM	226	[80]
GQDs	chemical oxidation	graphite fibers	Photoluminescent sensor	0–0.20 mM	330	[81]
GQD-B	chemical oxidation	carbon nano-onions	Photoluminescent sensor	20–200 nM	20	[82]
Eu-GQDs	strong acid cutting	3D Eu-graphene	Fluorescent probe	0.1–10 µM	56	[83]
GQDs@GSH	pyrolysis	citric acid/glutathione	Dual-photoluminescent probe	0.1–1.0 µM	53	[84]
T-GQDs	electrochemical oxidation	graphene film/K_2_S_2_O_8_	Fluorescent probe	0–20 µM	2000	[85]
GQDs	chemical oxidation	3D nanomesh graphene	Fluorescent sensor	0.1–1.0 µM	67	[86]

^1^ where LOD is limit of detection. af: amino-functionalized, B: boron, Eu: europium, GO: graphene oxide, GSH: glutathione, T: treated.

**Table 6:** The GQDs-based optical sensor for silver ion detection.

Type of GQDs	Synthesis Method	Starting Materials	Optical Method	Linear Range	LOD ^1^ (nM)	References
AgNPs/GQDs	microwave-assisted	GO nanosheets	Fluorescent sensor	0–100.0 nM	3.5	[90]
Am-GQDs	hydrothermal	dead leaves (carbon powder)	Photoluminescent probe	-	300,000	[91]
N-GQDs	microwave-assisted hydrothermal	glucose/ammonia	Fluorescent probe	0.2–40.0 µM	168	[92]
S-GQDs	hydrothermal	1,3,6-trinitropyrene	Fluorescent probe	0.1–130.0 µM	30	[93]
GQDs	pyrolysis	citric acid	Fluorescent probe	0.5–10.0 µM	180	[94]
GQDs	purchased	-	Ratiometric fluorescence sensor	0–115.2 µM	250	[96]

^1^ where LOD is limit of detection. AgNPs: Ag nanoparticles, Am: amine-terminated, GO: graphene oxide, N: nitrogen, S: sulfur.

**Table 7:** The optical sensors based on GQDs for the detection of other toxic metal ions.

Type of GQDs	Synthesis Method	Starting Materials	Optical Method	Metal Ion	Linear Range	LOD ^1^ (nM)	References
B-GQDs	electrochemical exfoliation	graphite rod	Fluorescent chemosensor	Al^3+^	0–100 µM	3640	[98]
N-GQDs	solvothermal	GO/dimethyl-formamide	Fluorescent probe	Al^3+^	2.5–7.5 µM	1300	[99]
gGQDs	microwave-assisted	GO nanosheets	ECL sensor	Cd^2+^	20–150 nM	13	[89]
TMPyP/NGQDs	hydrothermal oxidize	nitrogen-doped graphene	Fluorescent sensor	Cd^2+^	0.5–8 µM	88	[100]
NGQDs	hydrothermal reduction	nitrogen-doped GO	ECL sensor	Co^2+^	1.0–70 µM	200	[101]
GQDs	chemical oxidation	carbon fibers	Photoluminescent sensor	Ni^2+^	0–90 µM	4100	[102]

^1^ where LOD is limit of detection. B: boron, ECL: electrochemiluminescence, GO: graphene oxide, N: nitrogen, TMPyP: 5,10,15,20-tetrakis(1-methyl-4-pyridinio) porphyrin tetra(p-toluenesulfonate).
